# The Influence of Oily Vehicle Composition and Vehicle-Membrane Interactions on the Diffusion of Model Permeants across Barrier Membranes

**DOI:** 10.3390/membranes11010057

**Published:** 2021-01-14

**Authors:** Omaima N. Najib, Gary P. Martin, Stewart B. Kirton, Michelle J. Botha, Al-Sayed Sallam, Darragh Murnane

**Affiliations:** 1 Institute of Pharmaceutical Sciences, Franklin-Wilkins Building, Kings College London, 150 Stamford Street, London SE1 9NN, UK; omaima_najib@hotmail.com (O.N.N.); gary.martin@kcl.ac.uk (G.P.M.); 2 International Pharmaceutical Research Centre, 1 Queen Rania Street, Amman 11196, Jordan; 3 Department of Clinical and Pharmaceutical Science, College Lane, University of Hertfordshire, Hatfield AL10 9AB, UK; s.b.kirton3@herts.ac.uk (S.B.K.); m.botha@herts.ac.uk (M.J.B.); 4 Al-Taqaddom Pharmaceutical Industries, Co. 29-Queen Alia Street, Amman 11196, Jordan; a.sallam@tqpharma.com

**Keywords:** silicone membrane, binary vehicles, selective sorption, penetration enhancement, membrane vehicle interaction

## Abstract

In many instances, one or more components of a pharmaceutical or cosmetic formulation is an oil. The aims of this study were two-fold. First, to examine the potential of preferential uptake of one oily vehicle component over another into a model barrier membrane (silicone) from blended vehicles (comprising two from the common excipients isohexadecane (IHD), hexadecane (HD), isopropyl myristate (IPM), oleic acid (OA) and liquid paraffin). Second, to study the effect of membrane-vehicle interactions on the diffusion of model permeants (caffeine (CF), methyl paraben (MP) and butyl paraben (BP)) from blended vehicles. Selective sorption and partition of some oils (especially IHD and IPM) at the expense of other oils (such as OA) was demonstrated to take place. For example, the membrane composition of IHD was enriched compared to a donor solution of IHD-OA: 41%, 63% and 82% IHD, compared to donor solution composition of 25%, 50% and 75% IHD, respectively. Pre-soaking the membrane in IHD, HD or LP, rather than phosphate buffer, enhanced the flux of MP through the membrane by 2.6, 1.7 and 1.3 times, respectively. The preferential sorption of individual oil components from mixtures altered the barrier properties of silicone membrane, and enhanced the permeation of CF, MP and BP, which are typically co-formulated in topical products.

## 1. Introduction

The passage of compounds through a biological membrane after topical application is divided into four main stages. First, the compound has to be liberated from the formulation. Next, it penetrates into the outermost skin layer and then permeates (or partitions) sequentially from one layer into another, and finally reaches the vascular system. Permeation of a molecule through a membrane is influenced mainly by its activity gradient across the membrane thickness, as well as by its mobility within the membrane. Should the topical vehicle not interact with the membrane, then the compound would be expected to diffuse across the membrane according to its thermodynamic activity [1]. However, if a vehicle component interacts with the membrane in some way, then it may alter the membrane’s physicochemical properties. This in turn is likely to lead to the changed permeation of a co-formulated compound through the modified membrane in comparison to the original, unmodified membrane (e.g., if the compound is delivered using a non-interacting vehicle). This could be as a consequence of inducing change in the partition of the compound into the vehicle-containing membrane or by a direct effect on the barrier properties of the membrane, resulting in the modulation of drug transport [2]. Therefore, in some cases, the delivery and penetration of the vehicle to the membrane can be as important as that of the drug itself.

Most biological membranes are lipophilic, but are complex since they are composed of one or more layers, and this generally leads to slow drug diffusion [3]. The complexity also leads to high variability between the diffusion data of penetrants, using either harvested or cultured skin samples. Therefore, in order to seek to understand the basic principles affecting the permeation of different model penetrants, it can be advantageous to use less complex membranes such as those that comprise synthetic polymers [4,5]. For example, silicone membranes have been extensively used to characterize interaction and passive drug transport due to: their ease of preparation; hydrophobic character; and the relatively high diffusivities of drugs through the core matrix [6]. In addition, such membranes have been employed to study the influence of topical and transdermal formulation components on solute transport as a means of mimicking the lipoidal component of the stratum corneum membrane [7], despite their failure in providing an analogous model for the complexity of the stratum corneum [8].

The topical application of oils and oil-based formulations in skin care and skin protection has been a common practice since ancient times, and is a strategy still widely used for cosmetics. Oleaginous formulations are also extensively used as emollients and as topical vehicles for drug delivery. A primary factor in selecting an oil for use on the skin has traditionally been ‘the feel’ of the product on the skin. However, other factors may play a substantial role in successful topical treatment. For example, the relative occlusivity on the skin is important for achieving an effective moisturizing function. Oils also have been reported to enhance the percutaneous absorption of topically applied drugs [9,10]. Oily vehicles have traditionally been viewed as inert carrier systems for active ingredients and membrane permeation enhancers. Nevertheless, we have demonstrated that oily vehicles also partition extensively into synthetic (silicone, high density polyethylene and polyurethane) membranes [11], despite the conventional belief that such vehicles are inert and non-interacting with barrier membranes. Consequently, the flux of a range of model permeants was shown to be significantly affected by the choice of oily vehicle in which the permeant was applied, despite all formulations being applied having unit thermodynamic activity (i.e., saturated solutions) [11]. As an example of relevance to the current study, the effect of vehicle choice on the transport of methyl paraben (MP), butyl paraben (BP) and caffeine (CF) across silicone membrane are given in Table 1 below. The data reported in Table 1 are adapted by recalculation from their original presentation [11] into the more familiar flux (μg cm^−2^h^−1^).

Previous studies have considered some of the effects of single oils on the diffusion of co-administered permeants [11,12,13]. However, considering the effect of combining two oils within a vehicle, applying that vehicle topically to the membrane and then considering the interaction of those oils with membranes and the possible effects on permeants presented in the formulation has not previously been studied. This is despite the common practice (especially within cosmetic formulations) to blend oily vehicles to enhance organoleptic properties. The preferential uptake of one formulation component in a mixture may lead to a modification in the diffusion of other components within the formulation, including the drug [13,14]. Little attention has also been given to the ability of the oils included in cosmetic and topical products to enhance the unwanted permeation of formulation ingredients, such as preservatives. In this study, the uptake of different oily vehicles and blends of oils into silicone membrane and its effect on the diffusion of model permeants was investigated. The model permeants MP, BP and CF were selected on the basis of their having a differing lipophilicity, but similarity in molecular weight.

## 2. Materials and Methods

### 2.1. Materials

The sources of materials employed in this study have been given previously [11,15]. All of the materials employed were obtained from commercial suppliers, and used as received.

### 2.2. HPLC Chromatographic Conditions for Model Permeants

The assay used to analyze the permeants has been described previously [11,15]. A Symmetry 5 µm BDS (C18), 150 X 4.6 mm (5 µm) (Waters, Milford, MA, USA) was used for all three permeants. The mobile phases employed were: (a) 35% *v*/*v* acetonitrile/65% phosphate buffer (50 mM KH_2_PO_4_ containing 1% *w*/*v* triethylamine, then adjusted to pH 3.5 with orthophosphoric acid) for MP; (b) 50% acetonitrile/50% phosphate buffer (50 mM KH_2_PO_4_ adjusted to pH 3.0 with orthophosphoric acid) for BP; and (c) 15% *v*/*v* acetonitrile/85% phosphate buffer (50 mM KH_2_PO_4_ adjusted to pH 3.0 with orthophosphoric acid) for CF.

### 2.3. Solubility Studies

Solubility studies were conducted in accordance with previously published methodology [11,15]. In brief, an excess amount of MP, BP or CF was shaken in the medium of interest for 48 h at a temperature of 32 °C. After filtration to remove undissolved material and appropriate dilution, the filtrate was assayed for permeant, using the appropriate assay. Solubility data are presented for consideration as Supplementary Material to this article (Figure S1).

### 2.4. Franz Cell Studies

The protocol for the Franz cell experiments using synthetic membranes is described in detail elsewhere [11]. Briefly, the donor compartment contained a saturated suspension of permeant and diffusion was monitored into 2 mL of receptor medium (phosphate buffer pH 7.0) at 32 °C. The silicone membrane (0.32 mm in thickness, obtained from Samco UK Ltd, Nuneaton UK) had been pre-soaked in receptor medium overnight. The receptor phase was sampled (200 μL) and assayed at appropriate time points over 6 h. The sample removed was immediately replaced with an equal volume of receptor phase, and the concentration of diffused permeant corrected for dilution and previous sample removal.

### 2.5. Uptake Studies

#### Membrane Weight

Uptake of vehicles into silicone membrane was determined gravimetrically. The silicone membranes (0.32 mm in thickness, obtained from Samco UK Ltd., Nuneaton, UK) were cut to size (the diameter being approximately 2 cm) and the samples were then immersed in vehicle in a sealed glass vial, and soaked overnight (approximately 17 h) in a temperature-controlled water bath set at 32 °C. The membranes were blotted dry with tissue paper and reweighed. The percentage weight difference *(%* ∆*W*) was calculated according to Equation (1)

(1) $$\%\;\Delta W\;=\;\frac{\left(W_{a}\;-\;W_{b}\right)}{W_{b}}\;\text{×}\;100$$

where *W_a_* is the weight of membrane after soaking and *W_b_* is the weight of membrane before soaking.

### 2.6. Quantification of Oil Uptake by Silicone Membrane from Oil Blends

The amount of oil taken up by the silicone membrane was quantified using a combination of measurement of the mass of oil sorbed into the membrane, with GC analysis (Section 2.7) of oily components where this was feasible, following solvent extraction of the membrane with heptane. It was possible to assay isohexadecane (IHD), hexadecane (HD) and isopropyl myristate (IPM) by GC assay.

When oils that could be assayed by GC were used, the reweighed membranes were subsequently soaked in 3 mL of heptane for 1 h, the latter solvent replaced hourly with 3 mL of heptane up to 3 h (total 9 mL after pooling) to extract any sorbed oils from the membrane. The entire volume of heptane was combined, and subjected to GC analysis for each of IHD, HD or IPM. A control experiment was performed by extracting a sample of the membrane that was not soaked in oil for 3 h in heptane, to determine whether there was possible interference in the oil assay due to any potential extractable material present in the membrane.

### 2.7. GC Analysis

The analytical method development and validation for the IPM/IHD and IHD/HD vehicles was carried out using a gas chromatograph model Varian CTC Analytic (Varian, Palo Alto (CA), USA) previously reported [11] and are summarized here in brief. A 0.53 mm × 30 m fused silica capillary column bonded with a 1.0 µm film of phase (G42) (Quadrex, Bethany (CT), USA) was employed with helium as the carrier gas and flame ionization detection. For HD/IPM, the analysis and validation were carried out using a Focus gas chromatograph with a TriPlus auto sampler (Thermo, Karlsruhe, Germany) with the column employed being a 0.53 mm × 30 m fused silica capillary column bonded with a 1.0 µm film (InertCap GL Sciences Inc., Tokyo, Japan). The analytical parameters used to carry out the GC quantification of the oil mixtures are shown in Table S1 (Supplementary Materials).

### 2.8. Calculation of Amount Absorbed

The percentage amount of any specific oil absorbed by silicone membrane from the oil blends was calculated according to Equation (2)

(2) $$\%\;m_{upt}=\frac{\left(m_{GC}\right)}{m_{T}}\times 100$$

where *m_upt_* is the amount of oil sorbed, *m_GC_* is the amount of oil quantified by GC and *m_T_* is the total amount of oil sorbed by silicone membrane.

Since oleic acid (OA) and liquid parafin (LP) could not be determined using GC analysis, the amount of these (i.e., LP or OA in the membrane from the blends) was calculated according to Equation (3)

(3) $$\mathrm{Amount}\;\mathrm{of}\;\mathrm{LP}\;\mathrm{or}\;\mathrm{OA}=\;M_{T}-M_{2GC}$$

where *M_T_* is the total amount of oil sorbed by silicone membrane and *M*_2*GC*_ is the amount of second oil quantified by GC.

The accuracy of the gravimetric method of oil uptake (using Equation (3)) was assessed by comparing the total mass change of the membrane following the incubation of the membrane with mixtures of IHD-IPM, IHD-HD and HD-IPM with the masses of these oils determined by GC after solvent extraction (using heptane).

### 2.9. Determination of Composition of the Oil in Donor Compartment

In order to determine the ratio of the oils remaining in the donor compartment after diffusion experiments, a silicone membrane was cut to an appropriate size and immersed overnight in phosphate buffer pH 7.0. On the following day, the receptor compartment of a Franz cell with a nominal receptor phase volume of 2 mL and a diffusional area of 0.65 cm^2^ was carefully filled with phosphate buffer (PB), after which the receptor temperature was maintained at 32 °C by immersion in a temperature-controlled water bath. The membrane was then allowed to equilibrate with the receptor fluid for 1 h, after which time 200 μL of 75:25, 50:50 or 25:75% *w*/*w* IHD/HD solution was introduced into the donor compartment. After 6 h, any oil remaining in the donor compartment was removed for assay. The sample was diluted appropriately with heptane and analyzed using the GC method described in Section 2.7. Equations (4) and (5) were employed to calculate the percentage ratio of the oil applied and the oil remaining in the donor compartment after 6 h

(4) $$\%m_{oila}=\frac{\left(m_{oila}\right)}{m_{Ta}}\times 100$$

(5) $$\%m_{oilr}=\;\frac{\left(m_{oilr}\right)}{m_{Tr}}\times 100$$

where *m_oila_* is the amount of one of the component oils applied (i.e., either IHD or HD), *m_Ta_* is the amount of both oils introduced into the donor compartment, *m_oilr_* is the amount of that component oil remaining after 6 h and *m_Tr_* is the total amount of oil remaining in the donor compartment (i.e., the total amount of HD + IHD applied).

### 2.10. Effect of Membrane Pre-Treatment with Oil on Permeant Diffusion

The donor suspension was prepared by adding an excess amount of MP to 6 mL PB pH 7.0. The membrane was cut into circular discs, immersed and soaked in the HD, IHD and LP overnight at 32 °C. As a control, a sample of the membrane was immersed and soaked in PB overnight, rather than oil. Diffusion experiments were carried out using calibrated (for volume) Franz cells with a receptor phase of 2 mL and a diffusional area of 0.65 cm^2^. The membrane was cut to the appropriate size (diameter of around 2 cm) and placed between the donor and the receptor compartments of the Franz cell. The receptor compartment was carefully filled with PB pH 7.0 and the cell placed on a stirring plate submerged in a water-bath maintained at 32 °C. A small Teflon-coated magnetic bar (around 5 mm in length) was included in the receptor compartment, such that stirring was maintained throughout the duration of the experiment. After allowing the membrane to equilibrate with the receptor fluid, 200 μL of the buffer suspension (the sample contained undissolved/suspended solid of MP at 32 °C) was added to the donor compartment. At appropriate time intervals, 200 μL samples were withdrawn from the receptor compartment and immediately replaced with an equal volume of fresh PB (pH 7.0). The cumulative amounts (per unit surface area of membrane) of MP which diffused across membrane were plotted against time (h). The slope of the linear plot was taken as the flux of MP permeation. Each experiment was conducted five times.

### 2.11. Calculation of Enhancement Ratio

Enhancement ratios (ER) were derived from Equation (6):

(6) ER = Js(E)/Js(C)

where *J*s(E) and *J*s(C) are the flux values of the MP following pre-treatment of the membrane with oils (enhanced) or PB (control), respectively. Results were expressed as the mean ± standard deviation (SD) of 4–5 determinations.

## 3. Results

### 3.1. Silicone Membrane Diffusion Studies from Oils and Oil Combinations

**Figure 1:**
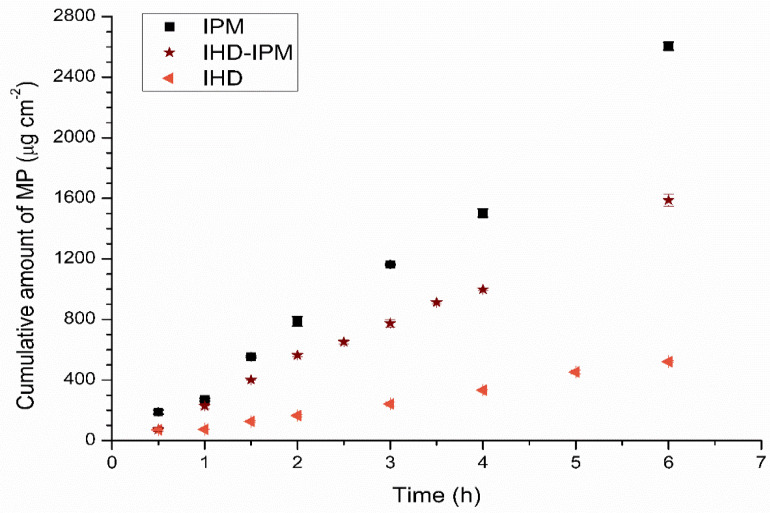
The cumulative amount of MP diffused across silicone membrane into the receptor compartment from the different vehicles as a function of time (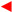) 100% IHD (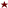) 50% HD–50% IPM, and (■) 100% IPM. Data represent mean ± sd (*n* ≥ 3). Error bars lie within the symbols.

The results obtained from the diffusion studies from different oil blends indicated that after a lag time, a linear correlation existed between the amount of penetrant accumulating in the receptor phase and time. The linear portion of the curve was used to calculate a flux. The correlation coefficients (R^2^), expressing the linearity of the plots were found to be ≥0.98 for the diffusion of permeants from all of the vehicles. Examples showing the permeation profiles for MP in 100% IHD, 50–50% IHD-IPM and 100% IPM across silicone membrane are depicted in Figure 1.

As shown in Table 1, the permeation of MP was affected by the choice of oily vehicle. However, it was evident from the results of the current study that the rate and extent of permeation of MP from a mixed oil system deviated from the anticipated effect of a linear change in composition for a saturated permeant solution in binary mixtures of IHD and IPM.

**Figure 2:**
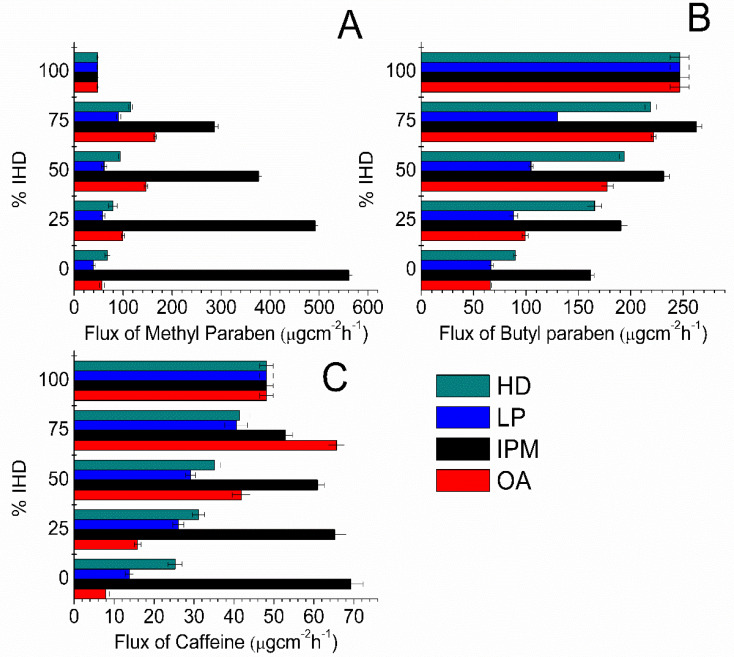
Flux values obtained for: (**A**) MP; (**B**) BP; and (**C**) CF diffusion through silicone membrane from different IHD oil combinations comprising IHD, HD, liquid paraffin (LP), IPM and OA. Data represent mean ± sd (*n* ≥ 3).

**Figure 3:**
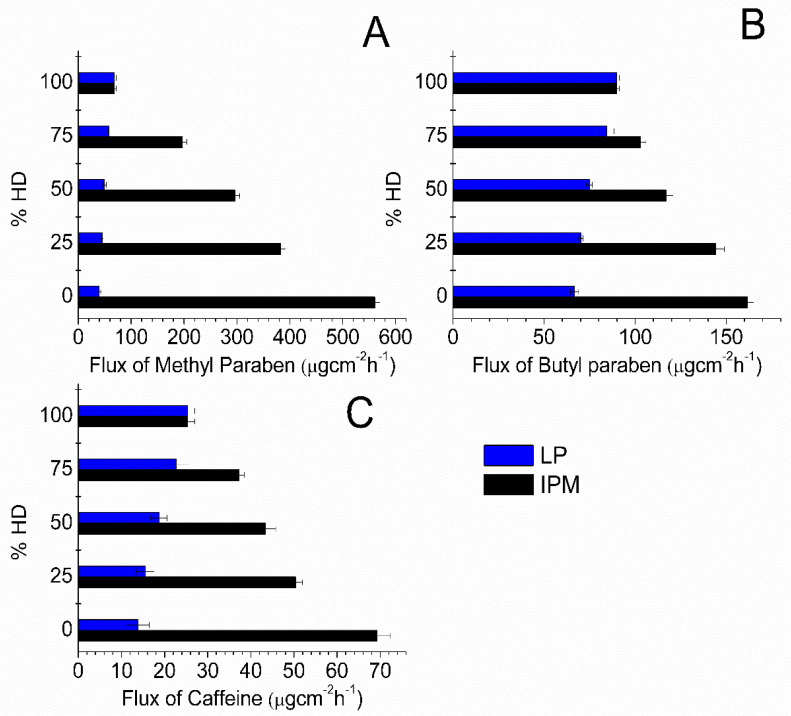
Flux values obtained for: (**A**) MP; (**B**) BP; and (**C**) CF diffusion through silicone membrane from different oil combinations comprising HD, LP and IPM. Data represent mean ± sd (*n* ≥ 3).

Since suspension systems were utilized, the equilibrium solubility was maintained throughout the experiments; however, the flux values were not constant when different oil blends were used. The influence of IHD content when present in binary mixtures of oil on the flux of MP, BP and CF is shown in Figure 2. Corresponding flux data for MP, BP and CF from HD binary blends are shown in Figure. 3. The highest fluxes were attained by blending IPM with IHD. The highest flux of MP and CF was from 100% IPM and the flux decreased as IHD or HD was added. However, the highest flux of BP was obtained when it was applied as a suspension in a 75% IHD/25% IPM mixture. The diffusion of MP from OA/IHD blends was at a maximum when suspended in a blend containing between 50–75% IHD (i.e., 50–25% OA) content (Figure 2A). With CF the maximum flux from OA/IHD mixtures was attained from a vehicle containing a blend of 75% IHD (25% OA) (Figure 2C). The flux from all oil blends (reported in Figure 2 and Figure 3) was higher than from the aqueous buffer (PB, Table 1) for all model permeants through silicone membrane; the fluxes from PB being 41.71 ± 1.01, 27.88 ± 0.46 and 13.01 ± 0.76 μgcm^−2^ h^−1^ for MP, BP and CF, respectively.

### 3.2. Quantification of Oil Uptake by Silicone Membrane from Oil Blends

#### 3.2.1. Change in Membrane Weight

**Figure 4:**
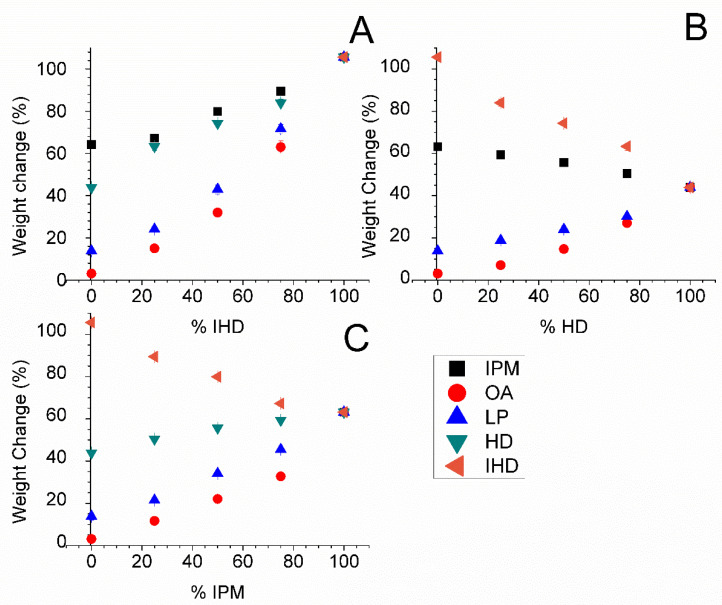
Percentage weight changes in silicone membrane immersed in different oil combinations comprising: (**A**) IHD; (**B**) HD; and (**C**) IPM with a second oil for approximately 17 h at 32 °C. Data represent mean ± sd (*n* ≥ 3). Error bars lie within the symbols.

Distinct differences were found in the weight increase of samples of silicone membrane when the latter were incubated with different combinations of oil in different ratios (Figure 4). IPM, IHD and HD were chosen as the base oils for admixture with LP and OA, since the three former oily vehicles, when used as single component vehicles, conferred the highest fluxes upon all three of the model permeants. The highest amount sorbed was when the membrane was incubated with IPM/IHD blends. On the other hand, the lowest amount of oil uptake was determined to occur from OA/HD blends.

#### 3.2.2. Measurement of Oil Uptake by Silicone Membrane

**Figure 5:**
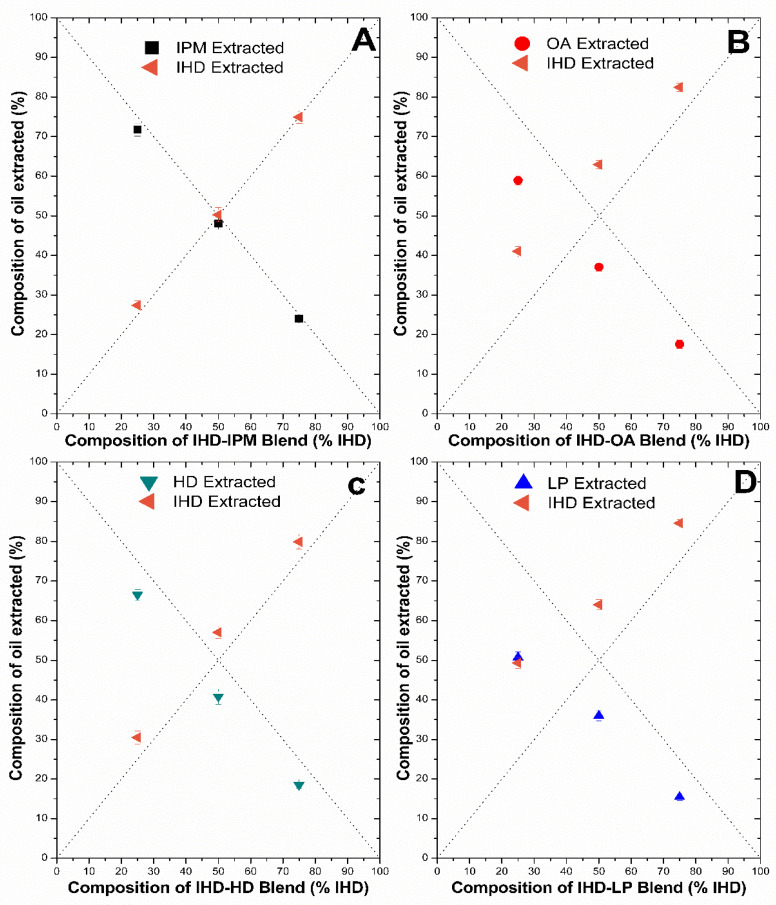
Percentage of individual oils extracted from silicone membrane after being incubated overnight at 32 °C in different blends of oil: IPM with IHD (**A**); OA with IHD (**B**); HD with IHD (**C**) and LP with IHD (**D**). Data represent mean ± sd (*n* ≥ 3). Error bars lie within the symbols.

**Figure 6:**
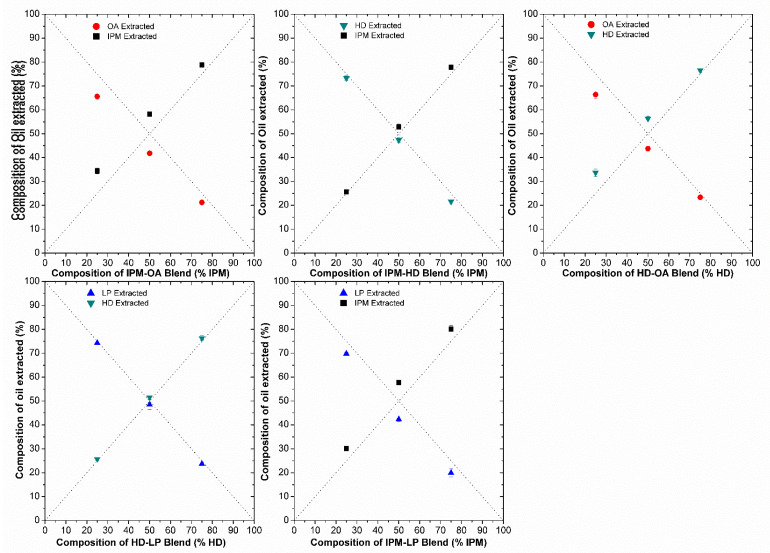
Percentage of individual oils extracted from silicone membrane after being incubated overnight at 32 °C in different blends of oil: OA with IPM (**A**); HD with IPM (**B**); OA with HD (**C**) LP with HD (**D**); and IP with IPM. Data represent mean ± sd (*n* ≥ 3). Error bars lie within the symbols.

The amount of oil taken up by silicone membrane was analyzed by GC chromatography. The quantity extracted from the membrane using heptane was then related to the ratio of oil originally present in the oil solution. The efficiency of determination of IHD, HD, IPM and their admixtures using the heptane extraction technique (described in Section 2.6) was ≥97%. The accuracy of extraction was therefore deemed sufficient to allow for the accurate estimation of LP and OA gravimetrically by difference using Equation (3), since no GC assay could be developed readily for these oils. Figure 5 and Figure 6 show the composition of oil extracted versus the composition of the oil blend at the start of incubation. Each plot includes the lines of identity (dashed lines), indicating the composition of the oil phase extracted from the membrane, for the scenario where the donor oils in the binary mixtures had been sorbed into the membrane in the same ratio as the mixture composition at the start of the membrane swelling experiments. The extraction results for IHD with other oils showed that IPM and IHD were sorbed to the same extent, and hence the composition of the amount of oil absorbed from the different ratio blends was close to the ratio present in the starting blend, and aligned on the line of identity. On the other hand, when IHD was blended with HD, LP or OA, the IHD appeared to be absorbed preferentially in comparison to the other oil present in the blend (with IHD consequently above its line of identity, and HD, LP and OA below their respective lines of identity). There was no significant difference in the amount of HD and IPM sorbed as a function of starting composition. However, when IPM was blended with either LP or OA then less of the latter two oils was sorbed by the membrane than was present in the original mixture. In all blends, the RSD between the samples was ≤5, which was deemed to be within acceptable limits for the GC method employed.

### 3.3. Determination of Composition of the Oil in Donor Compartment

**Figure 7:**
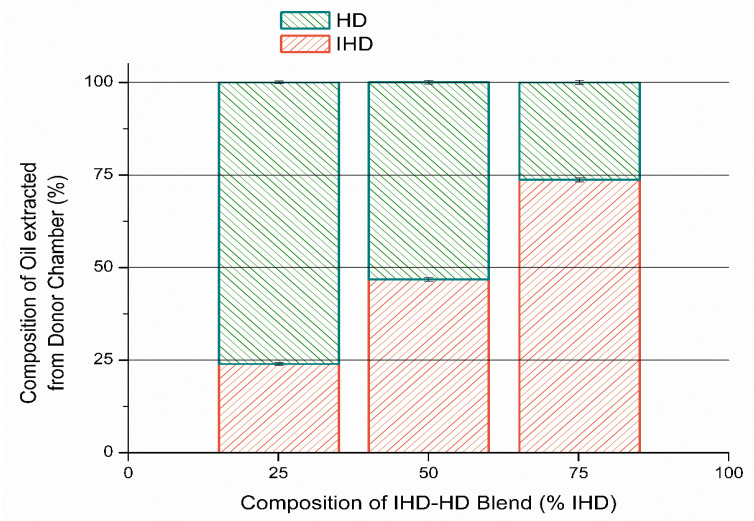
Percentage of individual oils (HD/IHD blends) remaining in the donor compartment after six hours, expressed as a function of the percentage of IHD applied in solution (mean ± SD).

Oil (200 µL) was added to the donor compartment of a Franz cell containing silicone membrane and a sample of the oil remaining in the donor compartment after 6 h (the duration of the diffusion experiments) was analyzed. The results in Figure 7 show the calculated percentage composition of oil applied and the percentage composition of oil remaining in the donor compartment after 6 h. These results indicate that the composition of the applied vehicle in the donor compartment changed significantly within the time that the experiment was conducted (*p* ≤ 0.05), although the magnitude of change in composition was not as large as the composition of the oil phase within the membrane. Nevertheless, the results did indicate that one oil was sorbed preferably compared to the other. Accordingly, therefore both the thermodynamic activity of any incorporated permeant might be altered, and the membrane structure or composition could be modified.

### 3.4. Effect of Membrane Pre-Treatment on Permeant Diffusion

In order to assess the effect of oils on silicone membrane, the membrane was soaked in LP, HD and IHD, and the diffusion of MP from buffer across such membranes was studied. These three oils were selected for study, since they were sorbed into the membrane in different ratios from oil binary blends. However, the three oils have similar solubility parameters and physicochemical characteristics, but comprise hydrocarbons that differ in their shape. MP was selected because it has relatively balanced hydrophilic/hydrophobic characteristics (i.e., an intermediate partition coefficient that is typical of many active pharmaceutical ingredients) and exhibits relatively high diffusivity through silicone membranes. Therefore, any pre-treatment solution that increased the MP permeation is likely to modify the permeation of other compounds [16]. The steady state flux of MP from phosphate buffer through silicone membrane pre-treated with different oils in comparison to a control comprising membrane pre-treated with buffer are shown in Table 2. Steady state fluxes were in the order of IHD > HD > LP. Generally, the flux of MP from pre-treated silicone membrane was found to be significantly higher than that of MP flux through silicone membrane soaked in buffer. These results indicate that the membrane is modified by the oil that has partitioned into the matrix, leading to the modification of the flux of drug presented at the same thermodynamic potential.

## 4. Discussion

Artificial membranes are often used during the development of a formulation to aid in the process of selecting a vehicle and excipients or alternatively as a quality control tool [17]. There are potentially a large number of bi-component mixtures that can be generated from the oils used in the current study, and indeed the oils are frequently present as mixed blends in pharmaceutical and cosmetic products. Since the potential array of oil blend (and oil blend-permeant) combinations available to choose for study were manifold, the effects of selecting only a few representative combinations on permeation of incorporated diffusants were examined.

The flux of a permeant across a non-porous barrier membrane is known to be directly proportional to its applied concentration as a consequence of the thermodynamic activity, or ‘leaving potential’ of the permeant [1]. Saturated systems were therefore used in this investigation to ensure equal thermodynamic activity of the permeant in the chosen vehicles, with excess solute being present so as to maintain saturation throughout the experiment. Thus, any drug lost from the solution by diffusion was replenished by dissolution of the excess drug. This methodology allows any effects of the vehicle on the diffusion of permeants through the membrane to be determined.

Partition of permeants from vehicles will be dictated by the creation of a more favorable environment for the increased solubility of the drug within the membrane. The latter would be mediated by donor solvents which are themselves well taken up into the membrane and also provide good solvency for the permeant within the membrane. The diffusion studies conducted using model permeants, showed that the highest fluxes of model permeants were obtained from pure IPM, IHD and blends of the two oils. MP and BP have a higher solubility in IPM and IPM/IHD combinations compared with the solubility in other oils (Figure S1, Supplementary Materials). The high solubility helps to quickly restore the amount of permeant lost by diffusion, and this keeps the thermodynamic activity constant. The enhanced permeation through the membrane might be due to the enhanced partitioning or diffusion of the compound through the membrane, or indeed a combination of both effects. The flux of model permeants from IPM/IHD combinations was in the order MP > BP > CF. This order is likely to be attributable to the relative magnitude of the partition coefficients, with MP more readily partitioning from oil to silicone membrane to buffer than the more hydrophobic BP or the more hydrophilic CF.

The enhancement of BP permeation from blends of IPM with IHD was shown to affect the diffusion in a manner that was not linearly dependent upon IHD content. A blend of 75% IHD in IPM was found to be the combination that gave the highest flux of BP. Similarly, the diffusion of MP and CF from OA/IHD blends formed parabolic curves with the peak in permeation occurring from blends containing 25% OA for CF; while with MP the maximum flux was attained from an oil mixture containing between 25–50% OA content. Blending IPM with HD led to lower flux results of the model permeants compared with those obtained for the same permeants dissolved in IPM/IHD blends. HD and IHD differ only in the structure and shape of the molecules. There was no marked difference in the solubilities of any given penetrant in 100% HD, IHD or LP (Figure S1, Supplementary material). There was also no marked differences between the solubility of a given permeant in IHD/IPM and HD/IPM blends. In the current study, the fluxes of BP from HD/IHD blends were higher than from the equivalent HD/IPM blends. In contrast, the fluxes of MP and CF were higher from HD/IPM blends than IHD/HD blends. These diffusion results along with the GC data show that this appears to be mainly due to the difference in the partitioning properties of the oils into the membrane, which might have led to modification of the membrane property, including the solubilizing capacity of oil-equilibrated membranes.

Diffusion studies of the model permeants were also carried out using the HD/LP and IHD/LP blends in order to investigate further the effects of a branched molecular structure and different molecular sized oil (LP) on the flux. The three oils are hydrocarbons with similar physicochemical properties, and there was no marked difference in the solubility of permeants in the IHD/LP and HD/LP blends (Figure S1, Supplementary Material). The viscosity of LP is higher than IHD and HD, and it has been reported that the viscosity of the vehicle does have an effect upon the release rate of a number of different dissolved solutes from the medium. The lower the viscosity of the vehicle the faster is the release [18]. However, the effect of viscosity of a vehicle on the diffusion rate of a permeant through a membrane might be minimal when compared to other potential factors, since most vehicles used are oils with similar viscosity. The flux of permeants depends mainly on the thermodynamic activity of the drug, and the partitioning properties of the drug and possibly vehicle components into the membrane. Since all the systems were thermodynamically uniform, it was expected that flux would be similar [1] from what are regarded as inert oily vehicles. The significantly higher flux of specific model permeants, through silicone membrane from the IHD/LP blends compared with the HD/LP blends, is likely to be primarily attributed to a difference in the vehicle partitioning into the membrane.

Binary mixtures of some vehicles have been reported previously to offer synergistic effects, in terms of leading to higher epidermal drug transport and reduced skin irritation [19,20]. If binary formulations exhibited a purely additive effect of the composite vehicles, in a non-interacting manner with the membranes, a direct, linear dependency of the transport of the permeants on the ratio (composition) of vehicle components would be expected. However, this has not proved to be the case for a number of transdermal formulations [21]. It has been shown previously that permeation of a molecule through a barrier membrane can occur independently of permeation of a co-applied molecule [2,22,23]. Nevertheless, preferential penetration of a specific vehicle component compared to the other component in a binary mixture has been shown in this work for the case of co-administered oils. Such differences between the composition and extent of uptake of vehicle components have not been reported previously, in the case of oily formulation bases.

When two oils are mixed and applied to a membrane, it has not been established previously whether the oils are sorbed in the same ratio as that in the applied solution. The results presented in Figure 5 and Figure 6 show the percentage amount of oil absorbed by silicone membrane from different oil blends can interact to different extents with silicone membrane. There was no significant difference in the total weight of oil sorbed when IHD was blended with either IPM or HD (Figure 4). However, when the partitioned oil was extracted and assessed by GC for its composition, there was a significant difference between the relative amount of IHD absorbed compared with HD (Figure 5). A similar finding was observed for sorption of IHD from mixtures of IHD/OA, and IHD/LP. IHD was absorbed into the silicone membrane in a greater proportion than the ratio present in the original applied blend. In contrast, the ratio of IHD/IPM sorbed from different ratios of the two oils was the same as that in the applied vehicle. Comparable results were obtained for HD/IPM blends (Figure 6). The amounts of IPM, IHD and HD extracted from the membrane were superimposable on the line-of-identity, and correlated linearly with the amounts of each in the original solution, from IPM/IHD and IPM/HD mixtures.

A study of the diffusion the behavior of linear alkanes, linear alcohols and substituted phenols in polyolefins, showed that as the number of carbon atoms increased, both the alcohols and phenols approached the behavior of the alkanes as a consequence of the increased shielding of the –OH group [24]. This finding was attributed to the high degree of rotational freedom about the carbon–carbon bond of the longer chains, leading to chains folding back upon themselves, as a consequence of intramolecular interactions involving the flexible methylene groups composing the carbon chain. These methylene moieties are able to form ‘ring’ structures with an adjacent moiety on the molecule (ester group) [25,26], while still retaining the linear structure. IPM could be behaving in the same way. IPM comprises a molecule containing a polar ester head group at the end of a relatively long hydrocarbon chain. As the number of carbon atoms is increased, the polarity is “diluted” by the presence of a longer hydrophobic tail, and this in turn leads to a lowering of the solubility parameter of any long ester. When blending IPM with HD or IHD, the partitioning behavior of the blends into a silicone membrane was the same, indicative of the weak specific interactions between silicone membrane and the ester group of IPM. The long alkyl chain attached to the ester group dominates the overall size and shape of the molecule, and diffusion occurs preferentially along the direction of greatest length of the molecule.

Application of blends of OA with IHD (Figure 5), HD (Figure 6) or IPM (Figure 6) showed that there were significant resultant differences in the amounts and the ratios of oleic acid sorbed to the membrane (compared to that present in the original applied blended oil). Due to the absence of a suitable GC assay method, the composition of OA in OA/IHD, OA/HD and OA/IPM blends within the membrane were determined by difference using Equation (3). However, the accuracy of the GC method was sufficient to enable an acceptable estimation of composition to be obtained by calculation. IHD appeared to be sorbed preferentially in comparison to both IPM and HD (in descending order) from blends of each with OA. From the values determined using the HSPIP software [11], OA has a solubility parameter (17.4 MPa^½^) that is similar to that of silicone membrane (17.4 MPa^½^). OA might have been expected to be taken up into the silicone membrane in greatest quantity; it was in fact the less preferentially sorbed oil from the different blends. This is likely to be a consequence of both the higher molecular weight of OA in comparison to other oils, as well as its larger molecular shape.

It has been reported that OA, in conjunction with polar solvents such as propylene glycol, is a more potent penetration enhancer on stratum corneum than when the former is employed alone [27]. The proposed explanation for this synergistic effect was the facilitated incorporation of OA into the lipid alkyl domains by the interaction of propylene glycol with the polar head groups [28]. It was also reported that short chained fatty acids were able to disrupt lipids when applied in a lipophilic mineral oil-based formulation, while the uptake of long chained fatty acids such as OA were enhanced when applied with hydrophilic, polar solvents such as propylene glycol [29]. In the current study blending IHD with OA enhanced the uptake of OA into the membrane. IHD swelled the silicone membrane, and such swelling could affect the packing of the cross-linked polymer chains, which might allow chain extension resulting in extra free volume, and facilitate OA uptake. This enhancement did not occur to the same extent as with HD or IPM blends with OA. Blending OA with HD, IPM and IHD produced the same trends where the amount of OA taken up by the membrane did not change between 50 and 75% (data not shown). The amount of LP sorbed by the membrane is low compared with the IPM, HD and IHD, possibly due in part to the content of LP molecules having a high molecular weight and a high molecular volume by comparison [30]. The higher molecular volume decreases the ability of the oil to be sorbed into the membrane. IPM and IHD were preferentially taken up by silicone membrane compared to LP, since they are smaller in size and have a higher affinity to the membrane.

The results show that there is a correlation between the amount of oil sorbed by the membrane and the flux of the model permeants. Oils which were sorbed by the membrane in greater amounts produced higher flux values for all model permeants regardless of the permeant tendency to partition into and diffuse through the membrane; this may suggest that oils have modified the properties of the membrane. In diffusion studies, the silicone membrane was soaked in buffer overnight, and then sandwiched between the donor and receptor chamber of the Franz cell. Oily vehicles were introduced into the donor compartment, while the receptor contained PBS. In order to determine the ratio of the oil remaining in the donor compartment under such conditions, blends of HD/IHD were added to the donor compartment, and a sample of the applied and remaining oils in the donor compartment were analyzed. HD/IHD blends were employed, since both oils have close physicochemical characteristics and the ratio of the two oils sorbed by silicone membrane from the blends was found to be significantly different in the uptake studies, to that present originally in the solution applied to the membrane. Furthermore, both oils could be readily detected and quantified by gas chromatography analysis. The results show that the composition of the oil was changed after 6 h. IHD was sorbed to a greater extent compared with HD over a period of six hours, therefore altering the original weight ratio. This indicated that since one oil was preferably sorbed compared to the other from this particular blended vehicle, then the thermodynamic activity of any applied drug in that vehicle would also be likely to be changed as a function of time. It would be either enhanced or reduced, depending on the drug solubility in the respective vehicle component and the amount of vehicle sorbed by the membrane.

Following differential sorption of mixed vehicle components into a membrane, the nature of the membrane might also change and possibly present an environment in which the agent is more soluble—promoting partition of the penetrant molecule into the membrane. Previous studies on the effect of pure penetration enhancers and mixtures on the barrier properties of the skin and concluded that a combination of two penetration enhancers was not necessarily more effective at enhancing transport than the individual components [21]. In the current study, OA was found to be the oily vehicle consistently sorbed onto/into the membrane to the lowest extent, and interestingly fatty acids were found to produce a significantly lower enhancement ratio compared to many other categories of ‘enhancer’ [21].

In order to assess the effect of oils on silicone membrane properties, the membrane was soaked in LP, HD and IHD, and the diffusion of MP from buffer across such membranes was studied. The pre-treatment of the silicone membrane with any of the oils was found to increase MP flux from all oils in comparison with the use of buffer (Table 2). The amount of oil sorption into silicone membrane correlated well with the enhancement ratio, the order of enhancement being IHD > HD > LP. The highest enhancement of IHD could be due to its higher sorption and presumably more favorable interactions with silicone membrane, leading perhaps to a greater diminution in its barrier properties. It might also (by its presence in the membrane) enhance the partition of MP into the oil-containing matrix of the membrane. It can be concluded from the results that as the amount of oil sorbed onto/into silicone membrane increases, the diffusion of the applied drug was promoted. Since the solubility of MP in IHD, HD and LP are broadly similar (range 1.21 ± 0.02–1.32 ± 0.03 mg mL^−1^), and the physicochemical properties of HD and IHD are the similar, it appears that the branching of IHD and the spherical shape of the molecule likely contributes to the positive enhancement in the uptake of MP and the latter’s subsequent penetration through the silicone membrane. Since the cumulative amount of MP that penetrated the silicone membrane was highest when the membrane was pretreated with IHD, this indicates that the simple oily vehicles did indeed alter membrane properties, as evidenced from the alteration of silicone membrane diffusional resistance.

In conclusion, it has been shown that oils (even those that are GRAS listed) have the potential to modify the barrier properties of synthetic membranes that are commonly used in pharmaceutical formulation screening studies. The modified membrane can affect ‘drug’ diffusion in two ways: (1) it acts to modify the barrier to the diffusion of many drugs; and (2) since the oils (from blends) may be taken up in different ratios, this could modify the composition of the applied vehicle such that the thermodynamic activity of the applied drug is altered, either within the vehicle, or within the membrane itself. The diffusion through synthetic membranes was found to be dependent upon the amount of oil sorbed by the membranes. Some of the oils that are currently widely used in cosmetics were found to enhance parabens’ permeation, compounds that are frequently used as preservatives in topical formulations. In addition, should the vehicle contain a blend of oils in the formulation, then these may be sorbed in different quantities to those present in the applied vehicle. Therefore, although some care should be exercised when developing blended products containing preservatives, judicious selection of oils may also facilitate a means to optimize drug uptake.

## 5. Declaration

This work constituted part of a larger thesis submitted in partial requirements for the degree of Doctor of Philosophy submitted to King’s College London by O.N.N. (22).

## Figures and Tables

**Table 1:** Flux of the methyl paraben (MP), butyl paraben (BP) and caffeine (CF) across silicone membrane form single component vehicles. (HD = hexadecane; IHD = isohexadecane; LP = liquid parafin; OA = oleic acid; IPM = isopropyl myristate; PB = phosphate buffer (pH 7.0)).

	MP Flux μg cm^−2^ h^−1^	BP Flux μg cm^−2^ h^−1^	CF Flux μg cm^−2^ h^−1^
HD	68.25 ± 1.39	89.83 ± 2.53	25.26 ± 1.72
IHD	135.5 ± 3.24	246.9 ± 5.34	48.15 ± 1.86
LP	39.12 ± 4.21	66.73 ± 1.26	13.79 ± 0.63
OA	56.67 ± 1.87	65.81 ± 2.29	7.92 ± 0.51
IPM	560.5 ± 7.92	161.63 ± 4.44	69.25 ± 4.10
PB	41.71 ± 1.01	27.88 ± 0.46	13.01 ± 0.76

**Table 2:** MP fluxes and enhancement ratios (ER) from buffer through silicone membrane pre-treated with IHD, HD, LP and buffer. Data are expressed as mean ± sd (*n* ≥ 4).

Pre-Treatment	Flux (μg cm^−2^ h^−1^)	ER
IHD	113.46 ± 1.83	2.62 ± 0.03
HD	75.60 ± 1.43	1.74 ± 0.03
LP	56.87 ± 1.99	1.31 ± 0.04
Buffer	43.25 ± 0.94	1.00 ± 0.03

## Data Availability

Not applicable.

## Supplementary Materials

The following are available online at https://www.mdpi.com/2077-0375/11/1/57/s1, Figure S1: Solubility (mg mL^−1^) in 100 % IHD, OA, IPM, LP, HD and different oil blends containing IHD or HD at 32°C (A) MP solubility in IHD blends (B)MP solubility in HD blends (C) BP solubility in IHD blends (D)BP solubility in HD blends (E) CF solubility in IHD blends (F)CF solubility in HD blends. Data represent mean sd (*n* ≥ 4). Error bars lie within the symbols, Table S1: Analytical parameters for the gas chromatographic quantification of IPM/IHD, IHD/HD and IPM/HD mixtures (IPM = isopropyl myristate; IHD = isohexadecane; HD = hexadecane).

## References

[1] Higuchi T. (1960). Physical Chemical Analysis of Percutaneous Absorption Process from Creams and Ointments. J. Soc. Cosmet. Chem..

[2] Dias M., Raghavan S., Hadgraft J. (2001). ATR-FTIR Spectroscopic Investigations on the Effect of Solvents on the Permeation of Benzoic Acid and Salicylic Acid through Silicone Membranes. Int. J. Pharm..

[3] Camenisch G., Folkers G., Van De Waterbeemd H. (1996). Review of Theoretical Passive Drug Absorption Models: Historical Background, Recent Developments and Limitations. Pharm. Acta Helvetiae.

[4] Watkinson A. (1995). The Influence of Vehicle on Permeation from Saturated Solutions. Int. J. Pharm..

[5] Russeau W., Mitchell J.C., Tetteh J., Lane M.E., Hadgraft J. (2009). Investigation of the Permeation of Model Formulations and a Commercial Ibuprofen Formulation in Carbosil® and Human Skin Using ATR-FTIR and Multivariate Spectral Analysis. Int. J. Pharm..

[6] Most C.F. (1970). Some Filler Effects on Diffusion in Silicone Rubber. J. Appl. Polym. Sci..

[7] Sloan K.B., Synovec J., Ketha H. (2013). A Surrogate for Topical Delivery in Human Skin: Silicone Membranes. Ther. Deliv..

[8] Moss G.P., Gullick D.R., Cox P.A., Alexander C., Ingram M.J., Smart J.D., Pugh W.J. (2006). Design, Synthesis and Characterization of Captopril Prodrugs for Enhanced Percutaneous Absorption. J. Pharm. Pharmacol..

[9] Kogan A., Garti N. (2006). Microemulsions as Transdermal Drug Delivery Vehicles. Adv. Colloid Interface Sci..

[10] Zhang A., Jung E.-C., Zhu H., Zou Y., Hui X., Maibach H. (2016). Vehicle Effects on Human Stratum Corneum Absorption and Skin Penetration. Toxicol. Ind. Heal..

[11] Najib O.N., Martin G.P., Kirton S.B., Sallam A.-S., Murnane D. (2016). Establishing the Importance of Oil-Membrane Interactions on the Transmembrane Diffusion of Physicochemical-LY Diverse Compounds. Int. J. Pharm..

[12] Karande P., Mitragotri S. (2009). Enhancement of Transdermal Drug Delivery via Synergistic Action of Chemicals. Biochim. et Biophys. Acta (BBA)—Biomembr..

[13] Haque T., Rahman K.M., Thurston D.E., Hadgraft J., Lane M.E. (2018). Topical Delivery of Anthramycin II. Influence of Binary and Ternary Solvent Systems. Eur. J. Pharm. Sci..

[14] McAuley W., Lad M., Mader K., Santos P., Tetteh J., Kazarian S., Hadgraft J., Lane M. (2010). ATR-FTIR Spectroscopy and Spectroscopic Imaging of Solvent and Permeant Diffusion across Model Membranes. Eur. J. Pharm. Biopharm..

[15] Najib O.N., Kirton S.B., Martin G.P., Botha M.J., Sallam A.-S., Murnane D. (2020). Multivariate Analytical Approaches to Identify Key Molecular Properties of Vehicles, Permeants and Membranes That Affect Permeation through Membranes. Pharmacy.

[16] Nanayakkara G.R., Bartlett A., Forbes B., Marriott C., Whitfield P.J., Brown M. (2005). The Effect of Unsaturated Fatty Acids in Benzyl Alcohol on the Percutaneous Permeation of Three Model Penetrants. Int. J. Pharm..

[17] Flynn G.L., Shah V.P., Tenjarla S.N., Corbo M., DeMagistris D., Feldman T.G., Franz T.J., Miran D.R., Pearce D.M., Sequeira J.A. (1999). Assessment of Value and Applications of In Vitro Testing of Topical Dermatological Drug Products. Pharm. Res..

[18] Yener G., Dal O., Üner M. (2009). Effect of Vehicles on Release of Meloxicam from Various Topical Formulations. Open Drug Deliv. J..

[19] Catz P., Friend D.R. (1990). Effect of Cosolvents on Ethyl Acetate Enhanced Percutaneous Absorption of Levonorgestrel. J. Control. Release.

[20] Chittenden J.T., Riviere J.E. (2015). Quantification of Vehicle Mixture Effects on in Vitro Transdermal Chemical Flux Using a Random Process Diffusion Model. J. Control. Release.

[21] Karande P., Jain A., Mitragotri S. (2006). Insights into Synergistic Interactions in Binary Mixtures of Chemical Permeation Enhancers for Transdermal Drug Delivery. J. Control. Release.

[22] Najib O.N. (2015). The Effect of Oil Structure on the Diffusion of Solutes across Synthetic Membranes and Human Epidermis. Ph.D. Thesis.

[23] McAuley W.J., Mader K.T., Tetteh J., Lane M.E., Hadgraft J. (2009). Simultaneous Monitoring of Drug and Solvent Diffusion across a Model Membrane Using ATR-FTIR Spectroscopy. Eur. J. Pharm. Sci..

[24] Koszinowski J. (1986). Diffusion and Solubility of Hydroxy Compounds in Polyolefines. J. Appl. Polym. Sci..

[25] Nisbet K., Harris F.W., Seymour R.B. (1977). Structure–Solubility Parameter Relationships in Alcohols. Structure-Solubility Relationships in Pol-Ymers.

[26] Forster S., Buckton G., Beezer A.E. (1991). The Importance of Chain Length on the Wettability and Solubility of Organic Homologs. Int. J. Pharm..

[27] Mahjour M., Mauser B.E., Fawzi M.B. (1989). Skin Permeation Enhancement Effects of Linoleic Acid and Azone on Narcotic Analgesics. Int. J. Pharm..

[28] Oh S., Jeong S.Y., Park T.G., Lee J.H. (1998). Enhanced Transdermal Delivery of Azt (Zidovudine) Using Iontophoresis and Penetration Enhancer. J. Control. Release.

[29] Wang M.Y., Yang Y.Y., Heng P.W.S. (2004). Role of Solvent in Interactions between Fatty Acids-Based Formulations and Lipids in Porcine Stratum Corneum. J. Control. Release.

[30] Postnov V.V., Gafarova N.A., Serikov Z.S., Nauruzov M.K., Malenko E.V. (1972). Composition of Liquid Paraffinic Hydrocarbons from Mangyshlak Crude. Chem. Technol. Fuels Oils.

