# Supplementary material for: The Influence of Oily Vehicle Composition and Vehicle-Membrane Interactions on the Diffusion of Model Permeants across Barrier Membranes
Source: Membranes (Basel). 2021 Jan 14;11(1):57. doi: 10.3390/membranes11010057 (PMC7830636; doi:10.3390/membranes11010057)
Supplement: Supplementary file 1 [file membranes-11-00057-s001.pdf]

membranes

Supplementary Material

The Influence of Oily Vehicle Composition and Vehicle-Membrane Interactions on the Diffusion of Model Permeants across Barrier Membranes

Omaima N Najib [1,2], Gary P Martin [1], Stewart B. Kirton [3], Michelle J. Botha [3], Al-Sayed Sallam [4] and Darragh Murnane [3,*]

1 Institute of Pharmaceutical Sciences, Franklin-Wilkins Building, Kings College London, 150 Stamford Street, SE1 9NN London, UK; omaima_najib@hotmail.com (O.N.N.); gary.martin@kcl.ac.uk (G.P.M.)
2 International Pharmaceutical Research Centre, 1 Queen Rania Street, Amman 11196, Jordan
3 Department of Clinical and Pharmaceutical Science, University of Hertfordshire, College Lane, AL10 9AB Hatfield, UK; s.b.kirton3@herts.ac.uk (S.B.K.); m.botha@herts.ac.uk (M.J.B.)
4 Al-Taqaddom Pharmaceutical Industries, Co. 29-Queen Alia Street, Amman 11196, Jordan; a.sallam@tqpharma.com
* Correspondence: d.murnane@herts.ac.uk; Tel.: +44-(0)1707-285904

Table S1: Analytical parameters for the gas chromatographic quantification of IPM/IHD, IHD/HD and IPM/HD mixtures (IPM = isopropyl myristate; IHD = isohexadecane; HD = hexadecane)

	**IPM/IHD**	**IHD/HD**	**IPM/HD**
Instrument temperature program			
Initial temperature (°C)	70	70	70
Initial time (min)	2	2	2
Rate (°C/ min)	7	8	7
Final temperature (°C)	260	295	260
Final time (min)	5	2	5
Injector temperature (°C)	220	220	220
Total time (min)	34.14	32.12	34.14
Inlet settings			
Mode split	Split	Split	Split
Split ratio	1:1	1:1	1:3
Injection volume (µL)	9.0	9.0	3
Flow rate (mLmin-1)	6	6	6

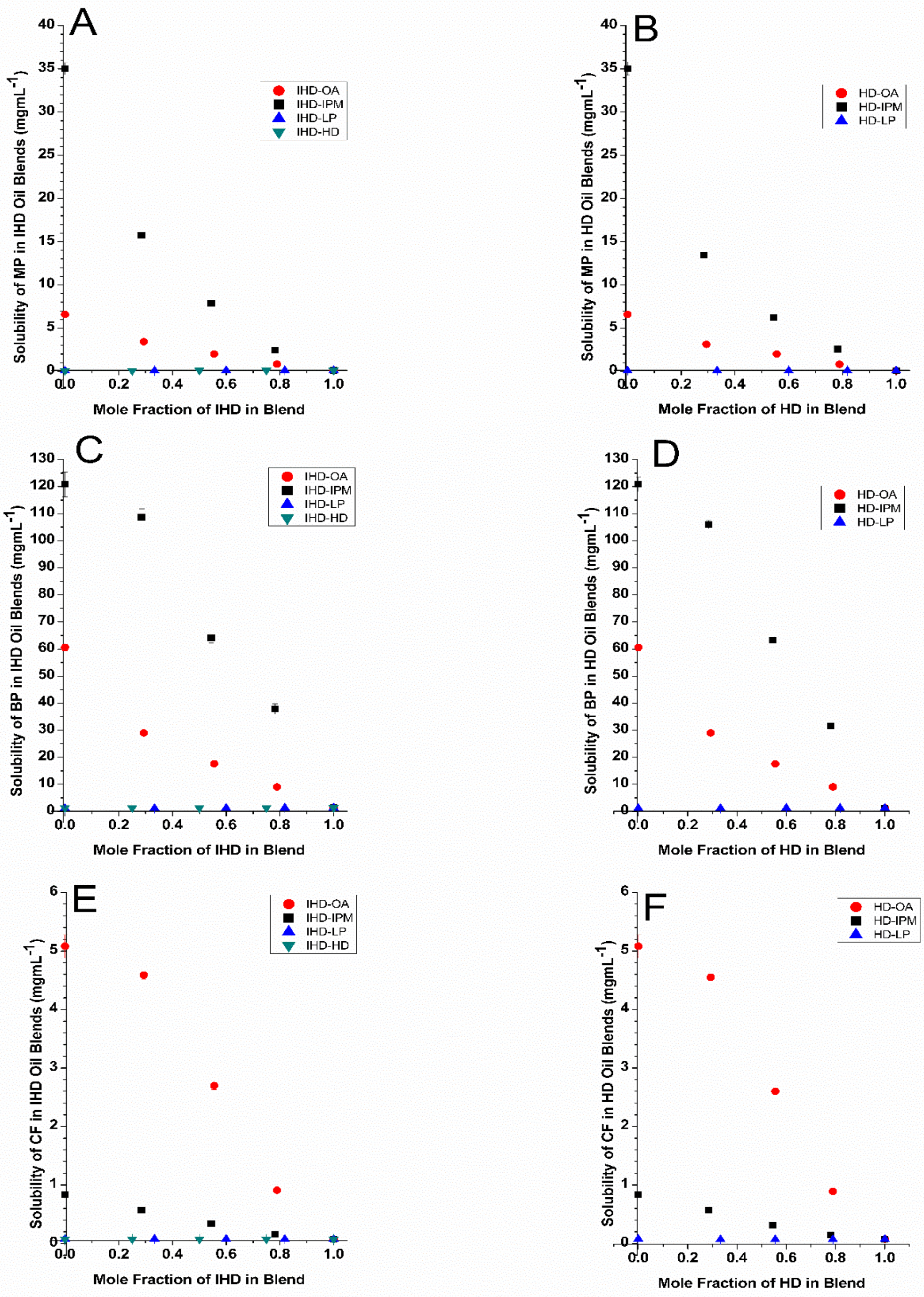

Figure S1. Solubility (mg mL^{-1}) in 100 % IHD, OA, IPM, LP, HD and different oil blends containing IHD or HD at 32 °C (**A**) MP solubility in IHD blends (**B**)MP solubility in HD blends (**C**) BP solubility in IHD blends (**D**)BP solubility in HD blends (**E**) CF solubility in IHD blends (**F**)CF solubility in HD blends. Data represent mean ± sd (n ≥ 4). Error bars lie within the symbols.
